# Validation of a measure to assess decision-making autonomy in family planning services in three low- and middle-income countries: The Family Planning Autonomous Decision-Making scale (FP-ADM)

**DOI:** 10.1371/journal.pone.0293586

**Published:** 2023-11-03

**Authors:** Jewel Gausman, Niranjan Saggurti, Richard Adanu, Delia A. B. Bandoh, Mabel Berrueta, Suchandrima Chakraborty, Ernest Kenu, Nizamuddin Khan, Ana Langer, Carolina Nigri, Magdalene A. Odikro, Veronica Pingray, Sowmya Ramesh, Paula Vázquez, Caitlin R. Williams, R. Rima Jolivet

**Affiliations:** 1 Women and Health Initiative, Department of Global Health and Population, Harvard University T.H. Chan School of Public Health, Boston, Massachusetts, United States of America; 2 Maternal and Child Health Nursing Department, School of Nursing, University of Jordan, Amman, Jordan; 3 Population Council, New Delhi, India; 4 Department of Population, Family and Reproductive Health, University of Ghana School of Public Health, Accra, Ghana; 5 Department of Epidemiology and Disease Control, University of Ghana School of Public Health, Accra, Greater Accra, Ghana; 6 Institute for Clinical Effectiveness and Health Policy, Buenos Aires, Argentina; 7 Department of Health Science, Kinesiology and Rehabilitation, Universidad Nacional de La Matanza, Buenos Aires, Argentina; 8 Department of Maternal and Child Health, Gillings School of Global Public Health, University of North Carolina at Chapel Hill, Chapel Hill, North Carolina, United States of America; Indian Institute of Dalit Studies (IIDS), INDIA

## Abstract

**Background:**

Integrating measures of respectful care is an important priority in family planning programs, aligned with maternal health efforts. Ensuring women can make autonomous reproductive health decisions is an important indicator of respectful care. While scales have been developed and validated in family planning for dimensions of person-centered care, none focus specifically on decision-making autonomy. The Mothers Autonomy in Decision-Making (MADM) scale measures autonomy in decision-making during maternity care. We adapted the MADM scale to measure autonomy surrounding a woman’s decision to use a contraceptive method within the context of contraceptive counselling. This study presents a psychometric validation of the Family Planning Autonomous Decision-Making (FP-ADM) scale using data from Argentina, Ghana, and India.

**Methods and findings:**

We used cross-sectional data from women in four subnational areas in Argentina (n = 890), Ghana (n = 1,114), and India (n = 1,130). In each area, 20 primary sampling units (PSUs) were randomly selected based on probability proportional to size. Households were randomly selected in Ghana and India. In Argentina, all facilities providing reproductive and maternal health services within selected PSUs were included and women were randomly selected upon exiting the facility. Interviews were conducted with a sample of 360 women per district. In total, 890 women completed the FP-ADM in Argentina, 1,114 in Ghana and 1,130 in India. To measure autonomous decision-making within FP service delivery, we adapted the items of the MADM scale to focus on family planning. To assess the scale’s psychometric properties, we first examined the eigenvalues and conducted a parallel analysis to determine the number of factors. We then conducted exploratory factor analysis to determine which items to retain. The resulting factors were then identified based on the corresponding items. Internal consistency reliability was assessed with Cronbach’s alpha. We assessed both convergent and divergent construct validity by examining associations with expected outcomes related to the underlying construct. The Eigenvalues and parallel analysis suggested a two-factor solution. The two underlying dimensions of the construct were identified as “Bidirectional Exchange of Information” (Factor 1) and “Empowered Choice” (Factor 2). Cronbach’s alpha was calculated for the full scale and each subscale. Results suggested good internal consistency of the scale. There was a strong, significant positive association between whether a woman expressed satisfaction with quality of care received from the healthcare provider and her FP-ADM score in all three countries and a significant negative association between a woman’s FP-ADM score and her stated desire to switch contraceptive methods in the future.

**Conclusions:**

Our results suggest the FP-ADM is a valid instrument to assess decision-making autonomy in contraceptive counseling and service delivery in diverse low- and middle-income countries. The scale evidenced strong construct, convergent, and divergent validity and high internal consistency reliability. Use of the FP-ADM scale could contribute to improved measurement of person-centered family planning services.

## Background

In its seminal 2001 report, “Crossing the Quality Chasm,” the United States Institute of Medicine defined six aims for quality of care, namely that care should be safe, effective, person-centered, timely, efficient, and equitable [1]. This framework was later adopted by the World Health Organization [2, 3]. More than a decade before, both Jain and Bruce proposed six dimensions for defining quality of care in the specific context of family planning, to include choice of methods, information given to clients, interpersonal relations, technical competence, continuity of care, and provision of an array of relevant services [4, 5]. Measuring quality of care in family planning services has likewise been the subject of decades of research [6–12] exploring various aspects of counseling to inform effective approaches [13].

More recently, integrating measures of respectful, person-centered care has been identified as an important priority in family planning, aligned with similar efforts in maternal care [6, 14]. Calls have been made for approaches that operationalize rights-based principles—such as respect, trust, empathy, privacy and confidentiality, and non-discrimination—into contraceptive counseling when assessing needs and preferences, providing information and support for decision making, and respecting clients’ choices [15]. Similarly, the principles of person-centered care focus on ensuring that individual preferences, needs, and values are at the forefront of all clinical decisions [16]. To respond to this need, a revision was proposed to Bruce’s original quality of care framework to better align it with rights-based approaches by specifically including aspects of dignity, respect, privacy, and confidentiality as part of client-provider interactions [17].

Historically, family planning programs have emphasized ensuring that a woman’s selection of a contraceptive method is an autonomous and informed choice, a priority aligned with basic human rights [18–20]. Ensuring that women have the ability to make autonomous decisions regarding their reproductive health is also a global priority highlighted in the direction-setting report for maternal mortality reduction in the Sustainable Development Goal (SDG) period, “Strategies toward Ending Preventable Maternal Mortality (EPMM)” [21], in its key theme related to the empowerment of women and girls. The report makes recommendations to empower women in the context of reproductive and maternal health care to ensure that they not only have decision-making power, but also the availability of options to allow them to exercise their choices. Subsequent work to prioritize a menu of the strongest available indicators to drive progress toward achieving the recommendations highlighted in the EPMM 11 Key Themes [22] identified, “proportion of women aged 15–49 who make their own informed decisions regarding sexual relations, contraceptive use, and reproductive health care” as one of the strongest existing indicators for applying a human rights framework to sexual, reproductive, maternal and newborn health care. Stakeholders to the process, however, called for modifications to the indicator to explicitly strengthen its ability to capture the construct of women’s decision-making power about timing and number of births as a measure of empowerment. Against this backdrop of efforts to strengthen available maternal health measures, a recent publication describes the development of a novel candidate indicator to measure the “proportion of women of reproductive age who make their own decisions about if, when, and how many children they want” [23].

In the field of maternal health, similar efforts to operationalize decision-making autonomy in measurement have led to the development and validation of measures focused on elements of person-centered care. The “Mothers Autonomy in Decision-Making (MADM)” scale was designed to measure autonomy in decision-making during maternity care, defined as a person’s ability to lead decision-making related to labor and childbirth, and focuses on person-led decision-making and client-provider discussions related to informed choice [24]. While other scales have been developed and validated in the field of family planning to measure other dimensions of person-centered care [25–28], none focus specifically on the construct of decision-making autonomy. For instance, the Interpersonal Quality of Care in Family Planning scale (IQFP) [27] focuses on primarily on measuring person-centeredness with regard to the interpersonal communication between provider and client, rather than focusing specifically on decisional autonomy. Similarly, the Quality of Contraceptive Counseling scale focuses on information exchange, interpersonal relationships, and disrespect and abuse [29]. To fill this gap, we have adapted the MADM scale to measure autonomy surrounding a woman’s decision to use a contraceptive method within the context of contraceptive counselling, developing the Family Planning Autonomous Decision-Making (FP-ADM) scale. The aim of this study was to conduct a psychometric validation of the FP-ADM scale using data from three diverse low- and middle-income countries: Argentina, Ghana, and India.

## Methods

### Study sample

This study used data obtained from a cross-sectional sample of women in four subnational geographic areas in Argentina, Ghana, and India. More information on the selection of the four study areas in the three countries can be found in the study protocol [30]. In each study area, participants were recruited using a two-staged, stratified random sample. In each subnational geographic area, 20 primary sampling units (PSUs) were randomly selected based on probability proportional to size.

### Ethics approval

The Institutional Review Board (IRB) of the Harvard T.H. Chan School of Public Health approved this study on 4 September 2019. The research is classified, using Harvard’s Data Security Policy, as Level 4 Data. The study also was approved in Argentina by the Comité de Ética de la Investigación de la Provincia de Jujuy (approval ID not applicable), Comisión Provincial de Investigaciones Biomédicas de la Provincia de Salta (approval ID: 321-284616/2019), Consejo Provincial de Bioética de la Provincia de La Pampa (approval ID not applicable), and Comité de Ética Central de la Provincia de Buenos Aires (approval ID: 2919-2056-2019); in Ghana by the Ghana Health Service Ethical Review Board (approval ID: GHS-ERC022/08/19); and in India by the national population council IRB (approval ID: 889) and local Sigma-IRB (approval ID: 10052/IRB/19-20). Written informed consent was obtained from all participants over the age of 18 years. The age of majority is 18 years old in Ghana, India, and Argentina; however, Argentine Civil Code says 16 years or older for those making decisions related to the care of their body. For indicators where women 15–49 will be surveyed, we will request parental consent and subject assent for participants aged between 15–17 years in Ghana and India, and aged 15 years old in Argentina.

### Data collection

Data were collected in Argentina between May and November 20201, in Ghana, between September 2020 and March 2021, and in India between October 2020 and March 2021. In Ghana and India, the sampling frame was derived from the most recent Demographic and Health Survey. A house listing exercise was undertaken in each selected primary sampling unit to identify households with a woman of reproductive age between the ages of 15 and 49 years. From the list, 18 women from different households in each primary sampling unit were randomly selected, for a total of 360 women per district and 1440 women per country. Interviews were conducted in the woman’s home. In Argentina, the sampling frame from the most recent Multiple Indicator Cluster Survey was used to randomly select primary sampling units, but a facility-based sample was used given the low population density. All facilities providing reproductive and maternal health services within the selected PSUs were included. Interviews were conducted with a random sample of 360 women per district upon exit from health facilities included in the study. In all three countries, women were included in this analysis if they agreed to participate, if they were aged between 15 and 49 years of age at the time of the study, and if they had ever received family planning services.

Data were collected on select sociodemographic variables, age including educational status, and literacy. Women were prompted to think about their most recent encounter with a family planning service provider. They were asked details about the types of services received from the provider, the quality of interpersonal care received during their visit using the 11 questions of the IQFP scale obtained from the validation study [27], and scale questions related to autonomous decision-making detailed below. Finally, women were asked about their current contraceptive use and future family planning intentions.

### Scale development and psychometric analysis

To measure the construct autonomous decision-making within the context of family planning service delivery, we adapted the seven items of the MADM scale [24] to focus on questions specific to family planning. The specific items included in the scale can be found in **Table 1**. For each question, women were asked to rate the health care provider they most recently saw for family planning services with respect to the items in the scale. Participants were then asked to respond to each item as 1 “Not at all,” 2 “Somewhat/a little bit,” and 3 “Yes, very much.”

**Table 1:** FP-ADM scale items.

Question	Item
1	The healthcare provider asked me how much information I wanted to receive about various methods of contraception
2	The healthcare provider told me that there are different options for my family planning
3	The healthcare provider explained the advantages and disadvantages of the family planning options
4	The healthcare provider helped me understand all the information
5	The healthcare provider gave me enough time to thoroughly consider the different family planning options
6	The healthcare provider let me choose what I considered to be the best family planning option for me
7	The healthcare provider respected that choice

Few respondents in any country were missing responses to any of the questions on the scale. Missing responses were assumed to be missing at random as no discernable patterns emerged after examining the missing responses across the three countries in the study. The largest number of missing responses to any item in the FP-ADM scale was 19 (2.14%) in Argentina, 2 (0.18%) in Ghana and 12 (1.06%) in India. Additionally, few respondents refused to answer specific questions on the scale; the highest number of refusals to any question being 3 (0.58%) in Argentina, 15 (1.35%) in Ghana, and 16 (1.42%) in India. These refusals resulted in dropping an additional 15 respondents in Argentina (1.69%), 38 in Ghana (3.41%), and 17 in India (1.50%). Only women with complete responses to scale items were retained for analysis. The final sample consisted of 890 women in Argentina, 1,114 in Ghana and 1,130 in India.

To assess the psychometric properties of the scale, we first examined the scale’s Eigenvalues and conducted a parallel analysis with ten replications to determine the number of factors to retain. We then conducted exploratory factor analysis with oblique (promax) rotation to determine which items to retain in the scale. Items were included in the final scale if they had factor loadings greater than 0.40. The resulting factors were then identified based on the corresponding items. Internal consistency reliability was assessed by calculating Cronbach’s alpha on the final scale and subscales.

### Validity testing

We assessed both convergent and divergent construct validity by examining associations with expected outcomes hypothesized to be associated with the underlying construct using t-tests. We expected that women with higher decision-making autonomy at their last family planning visit would be more likely to 1) be using their preferred contraceptive method, 2) have higher satisfaction with their current method, and 3) want to continue using their method until a pregnancy is desired. Further, we hypothesized that women with higher decision-making autonomy would have higher overall satisfaction with their visit. Overall satisfaction was measured by asking women, “Are you satisfied with the quality of care you received from the health or family planning worker?” Possible responses were “Not at all” (1), “Somewhat/a little bit” (2), and “Yes, very much” (3). We assessed convergent validity by examining whether there was a positive association observed between women’s IQFP scores and FP-ADM scores. Given that both the IQFP and FP-ADM scales examine different dimensions related to person-centered care, we expected to see a positive association between the two conceptually related measures. To assess divergent validity, we explored whether a woman’s FP-ADM score was negatively associated with planned method discontinuation by her indicating a desire to switch to another method in the future. Given the high usage of sterilization in India, we also performed a sensitivity analysis on the Indian sample in which we excluded women who were using sterilization.

### Inclusivity in global research

Additional information regarding the ethical, cultural, and scientific considerations specific to inclusivity inn global research is included in the S1 Checklist.

### Findings

The characteristics of the study sample are presented in **Table 2**. In all three countries, the largest proportion of women was between 25 and 34 years of age, with the population skewing slightly older in India and slightly younger in Argentina and Ghana. In Argentina and India, the majority of women had secondary or higher education (76.26% in Argentina and 45.01% in India) and could read and write (98.71% in Argentina and 75.29% in India). In Ghana, a large proportion of participants had no formal education (39.78%) and over half could not read or write (55.58%). In Argentina, nearly one-quarter of the study sample was pregnant (23.97%) compared to 12.92% in Ghana and 5.39% in India.

**Table 2:** Description of study sample.

	Argentina	Ghana	India
	n (%)	n (%)	n (%)
Sample Size	851	1076	1113
Age			
15–24 years	303 (35.61)	299 (27.79)	165 (14.82)
25–34 years	364 (43.95)	428 (39.78)	520 (46.72)
≥35 years	173 (20.33)	349 (32.43)	428 (38.45)
Missing/refused	1 (0.12)	0 (0.0)	0 (0.0)
Educational attainment			
No formal education	4 (4.47)	428 (39.78)	109 (9.79)
Primary education	196 (23.03)	341 (31.69)	356 (31.99)
Secondary or higher	649 (76.26)	299 (27.79)	501 (45.01)
Missing/refused	2 (0.24)	8 (0.74)	147 (12.31)
Literacy			
Read and write	840 (98.71)	430 (39.96)	838 (75.29)
Read only	8 (0.94)	28 (2.60)	5 (0.45)
Sign only	2 (0.24)	14 (1.30)	133 (11.95)
Cannot read or write	0 (0.0)	598 (55.58)	136 (12.22)
Missing/refused	1 (0.12)	6 (0.56)	1 (0.09)
Currently Pregnant			
Yes	204 (23.97)	139 (12.92)	60 (5.39)
No	641 (75.32)	936 (86.99)	1053 (94.61)
Don’t know	6 (0.71)	1 (0.09)	0 (0.0)
Current Method Use*			
Not using any method	69 (12.32)	133 (27.48)	35 (3.99)
Female Sterilization	83 (9.75)	1 (0.22)	423 (50.24)
Intra-uterine Device (IUD)	75 (8.81)	9 (2.01)	42 (4.99)
Implant	63 (7.40)	71 (15.85)	0 (0.00)
Injectable	68 (7.99)	158 (35.27)	23 (2.73)
Oral Contraceptive Pill	134 (15.75)	41 (9.15)	63 (7.48)
Male Condom	111 (13.04)	23 (5.13)	288 (34.20)
Female Condom	0 (0.00)	0 (0.00)	5 (0.59)
Emergency Contraception	3 (0.35)	10 (2.23)	4 (0.48)
Diaphragm	0 (0.00)	0 (0.00)	2 (0.24)
Foam/Jelly	0 (0.00)	0 (0.00)	6 (0.71)
Contraceptive Patch	1 (0.12)	0 (0.00)	0 (0.00)
Standard Days Method	3 (0.35)	33 (7.35)	10 (1.19)
Lactational Amenorrhea Method	13 (1.53)	13 (2.90)	1 (0.12)
Other Modern Method	4 (0.47)	1 (0.22)	1 (0.12)
Withdrawal	7 (0.82)	6 (1.34)	21 (2.49)
Fertility Awareness/Periodic Abstinence	1 (0.12)	7 (1.56)	1 (0.12)
Other Traditional Method	0 (0.00)	1 (0.22)	0 (0.00)
Don’t know/Refused	4 (0.47)	2 (0.45)	0 (0.00)

*May add up to more than 100% as multiple response categories for methods were allowed.

Most non-pregnant women in the study were using a method of contraceptive (83.85% in Argentina, 66.13% in Ghana, and 93.1% in India). The pill and male condom were the most commonly used methods in Argentina (13.81% and 11.39%, respectively), while in Ghana, the most commonly used methods were injectables (33.95%) and implants (15.11%). Half of the women in India were using female sterilization compared to 8.31% in Argentina and 0.21% in Ghana.

The content of the women’s most recent encounter with family planning services is detailed in **Table 3**. In Ghana and India, >90% of women indicated that their most recent family planning visit included general family planning counseling and education, compared to only 69.10% in Argentina. Approximately 25% of the population in Argentina and India received method specific counseling and education, compared to 35.13% in Ghana. More than 30% of women in Argentina received method provision compared to only 13.94% in Ghana and 1.71% in India.

**Table 3:** Characteristics of most recent family planning services, percent of women.

	Argentina	Ghana	India
	n (%)*	n (%)*	n (%)*
Sample Size (n)	851	1076	1113
General family planning counseling and education	588 (69.10)	974 (90.52)	1088 (97.75)
Method specific counselling and education	197 (23.15)	378 (35.13)	297 (26.68)
Received written information only	127 (14.92)	44 (4.09)	11 (0.99)
Method provision	284 (33.37)	150 (13.94)	19 (1.71)
Don’t know	3 (0.35)	1 (0.09)	2 (0.18)
Refused	7 (0.82)	2 (0.19)	4 (0.36)

*May add up to more than 100% because multiple responses were allowed

Women’s responses to individual scale items are presented in **Table 4**. Across all three countries, the smallest percentage of women gave their provider a score of 3 (“yes, very much”), to the question asking whether the provider asked how much information the woman wanted to receive about the various methods of contraception (item 1). For each of the remaining six items on the scale, >80% of participants in Argentina and Ghana rated the provider with the highest score possible of 3 (“yes, very much”). Scores tended to be lower in India, with a much higher percentage of women giving their healthcare provider a rating of 2 (“somewhat/a little bit”) than in the other two countries. In India, between 36% and 55% of women responded with a score of 2 (“somewhat/a little bit”) to each survey item, compared to < 15% in Argentina and Ghana. Few women in any country gave their health care provider a rating of 1 (“no, not at all”) to any question.

**Table 4:** Women’s responses to individual items on the FP-ADAM scale.

		Argentina	Ghana	India
The healthcare provider asked me how much information I wanted to receive about various methods of contraception	No, not at all (1)	202 (23.74)	70 (6.51)	80 (7.19)
	Somewhat/a little bit (2)	111 (13.04)	126 (11.71)	609 (54.72)
	Yes, very much (3)	536 (63.22)	880 (81.78)	424 (38.10)
The healthcare provider told me that there are different options for my family planning	No, not at all (1)	65 (7.64)	32 (2.97)	148 (13.30)
	Somewhat/a little bit (2)	47 (5.52)	83 (7.71)	409 (36.75)
	Yes, very much (3)	739 (86.84)	961 (98.31)	556 (49.96)
The healthcare provider explained the advantages and disadvantages of the family planning options	No, not at all (1)	91 (10.69)	65 (6.04)	132 (11.86)
	Somewhat/a little bit (2)	65 (7.64)	116 (10.78)	461 (41.62)
	Yes, very much (3)	695 (81.67)	895 (83.18)	520 (46.72)
The healthcare provider helped me understand all the information	No, not at all (1)	44 (5.17)	52 (4.83)	83 (7.46)
	Somewhat/a little bit (2)	7.64 (65)	131 (12.17)	448 (40.25)
	Yes, very much (3)	742 (87.19)	893 (82.99)	582 (52.29)
The healthcare provider gave me enough time to thoroughly consider the different family planning options	No, not at all (1)	90 (10.58)	58 (5.39)	75 (6.74)
	Somewhat/a little bit (2)	83 (9.75)	102 (9.48)	537 (48.25)
	Yes, very much (3)	678 (79.67)	916 (85.13)	501 (45.01)
The healthcare provider let me choose what I considered to be the best family planning option for me	No, not at all (1)	59 (6.93)	25 (2.32)	59 (5.30)
	Somewhat/a little bit (2)	47 (5.52)	81 (7.53)	459 (41.24)
	Yes, very much (3)	745 (87.54)	970 (90.15)	595 (53.46)
The healthcare provider respected that choice	No, not at all (1)	39 (4.58)	18 (1.67)	44 (3.95)
	Somewhat/a little bit (2)	32 (3.76)	62 (5.76)	415 (37.29)
	Yes, very much (3)	780 (91.66)	996 (92.57)	654 (58.76)

**Fig 1:**
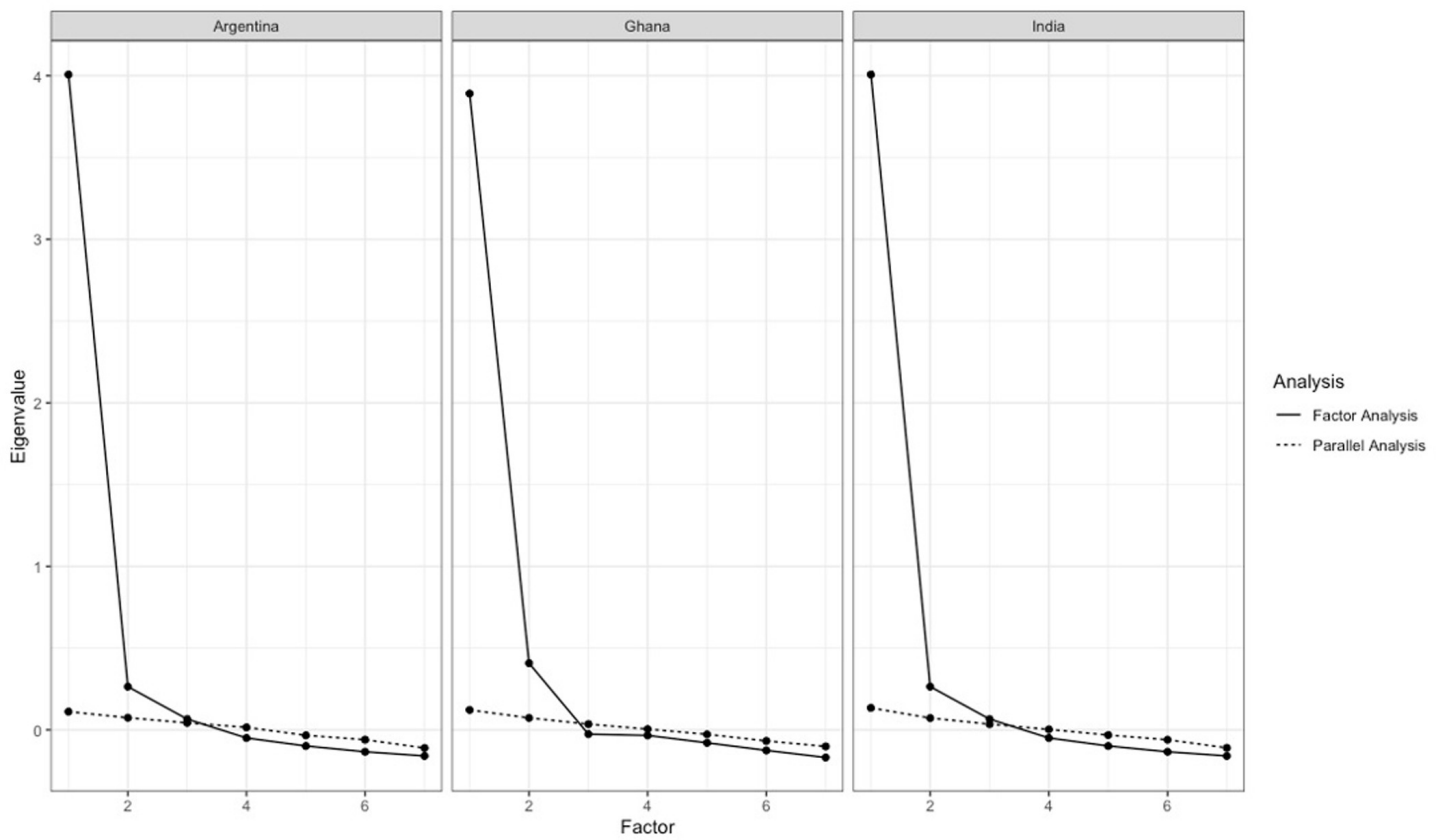
Scree plot and parallel analysis of FP-ADAM scale.

The Eigenvalues and parallel analysis suggested a two-factor solution. The scree plot of the Eigenvalues obtained from the parallel analysis in each country is presented in **Fig 1**. A clear elbow is present at a two-factor solution. The parallel analysis also indicated that there were at least two factors represented in the construct, with the possibility of a third given that the Eigenvalue of the third factor was very close in value to the average Eigenvalue for the third random factor obtained from the parallel analysis. Factor loadings for both a two- and three-factor solutions favored a two-dimensional construct as no items clearly loaded on the third factor in any country (**Table 5**). In all three countries, scale items 1–5 clearly loaded onto the first underlying factor, while scale items 6–7 clearly loaded onto the second underlying factor. All factor loadings were above the threshold of 0.4, except for scale item 2 in Ghana, which did not uniquely load onto either factor. The two underlying dimensions of the construct were identified as “Bidirectional Exchange of Information” (factor 1) and “Empowered Choice” (factor 2). Cronbach’s alpha was calculated for the full scale and each subscale corresponding to the two identified factors (**Table 6**). In all cases, Cronbach’s alpha was >0.7 suggesting that the full scale and subscales have good internal consistency reliability. There were no appreciable differences noted in the factor loadings or Cronbach’s alpha after removing women using sterilization in India. Cronbach’s alpha was 0.9 on the full scale, and 0.8 and 0.7 on subscale 1 and 2, respectively.

**Table 5:** Factor loadings for FP-ADM scale (two-factor solution).

	Argentina		Ghana		India	
	Factor 1	Factor 2	Factor	Factor 2	Factor	Factor 2
Scale Items						
The healthcare provider asked me how much information I wanted to receive about various methods of contraception	**0.59**	0.01	**0.71**	0.13	**0.79**	0.01
The healthcare provider told me that there are different options for my family planning	**0.62**	0.02	0.38	0.39	**0.70**	0.13
The healthcare provider explained the advantages and disadvantages of the family planning options	**0.73**	0.01	**0.82**	-0.02	**0.71**	0.11
The healthcare provider helped me understand all the information	**0.62**	0.16	**0.85**	0.00	**0.57**	0.31
The healthcare provider gave me enough time to thoroughly consider the different family planning options	**0.61**	0.20	**0.56**	0.27	**0.53**	0.24
The healthcare provider let me choose what I considered to be the best family planning option for me	0.15	**0.77**	0.07	**0.72**	0.13	**0.67**
The healthcare provider respected that choice	-0.02	**0.84**	-0.02	**0.78**	0.06	**0.72**

**Table 6:** Cronbach’s alpha for each sub-scale and FP-ADM scale.

	Argentina	Ghana	India
Information and Education Subscale (factor 1)	0.81	0.89	0.89
Choice Subscale (factor 2)	0.87	0.79	0.79
Full FP-ADAM Scale	0.85	0.89	0.9

The mean FP-ADM score was 2.72 (SD 0.44) in Argentina, 2.82 (SD: 0.37) in Ghana, and 2.41 (SD: 0.50) in India. The mean score in India was 2.34 (SD: 0.2) after removing women who were using sterilization from the sample. **Table 7** presents the associations between the variables related to contraceptive use, contraceptive continuation, and satisfaction with family planning services that were hypothesized to be related to the construct of decisional autonomy. In Argentina and Ghana, the FP-ADM scale had a significant positive association with current use of preferred method, satisfaction with current method, and a woman’s intention to continue using the method until a pregnancy is desired. In India, no significant associations were observed between the FP-ADM scale and current use of preferred method and a woman’s intention to continue using the method until a pregnancy is desired, though the difference in the mean scores were in the expected direction. The lack of statistical significance is likely the result of there being very few women stating that they were not using their preferred method. These findings did not change when women using sterilization were removed from the sample, and likely due to the small number of women (n = 4) who stated that they were not using their preferred method or that they wanted to switch.

**Table 7:** Associations with FP-ADM scale and preferred method use, method satisfaction, and satisfaction with family planning service provision.

	Argentina				Ghana				India			
	n	Mean	SD	p	n	Mean	SD	p	n	Mean	SD	p
Currently using preferred method												
No	96	2.67	0.5	0.002	23	2.65	0.54	0.002	4	2	0.61	0.111
Yes	338	2.8	0.36		323	2.87	0.3		838	2.4	0.5	
Satisfied with current method												
No	89	2.63	0.47	<0.0001	53	2.65	0.49	<0.0001	54	2.74	0.41	<0.0001
Yes	323	2.82	0.36		387	2.9	0.28		787	2.39	0.49	
Want to continue current method until pregnancy is desired												
No	67	2.6	0.52	<0.0001	40	2.63	0.5	<0.0001	4	2.21	0.83	0.462
Yes	354	2.82	0.33		308	2.89	0.28		837	2.4	0.5	
Satisfied with quality of care received from healthcare provider												
No	86	1.83	0.52	<0.0001	127	2.16	0.51	<0.0001	491	2.07	0.4	<0.0001
Yes	763	2.83	0.29		946	2.91	0.23		622	2.69	0.39	

As expected, there was a strong and significant positive association between a woman expressing satisfaction with the quality of care received from the healthcare provider and a woman’s score on the FP-ADM score in all three countries. In Argentina women who reported being satisfied with the quality of care received from their healthcare provider scored a full point higher on the FP-ADM scale (1.83 versus 2.83; p<0.0001). In Ghana and India, the difference in score between women who reported being satisfied with the quality of care was not as high of a magnitude as observed in Argentina (0.75 points different in Ghana and 0.62 points different in India; p<0.0001 for both comparisons). As expected, we also found a significant negative association between a woman’s FP-ADM score and her desire to switch to another method in the future in all three countries.

When comparing the performance of the FP-ADM scale to the IQFP, we found a significant positive association between a respondent’s mean IQFP and FP-ADM scores. For each one-point increase in a respondent’s mean FP-ADM score, there was an average increase of IQFP score by 0.60 point in Argentina (95% CI: 0.56–0.63; p<0.0001), 0.79 point in Ghana (95% CI: 0.75–0.82; p<0.0001) and 0.80 point in India (95% CI: 0.78–0.83; p<0.0001). In India, after removing women who were using sterilization, the results were very similar to those of the full sample, with an average increase of IQFP of 0.80 point (95%: CI 0.78–0.83; p<0.0001).

## Discussion

Our results suggest that the FP-ADM is a valid instrument to assess decision-making autonomy in the context of contraceptive counseling and service delivery in diverse low- and middle-income countries.

The psychometric properties of the scale evidenced strong construct, convergent, and divergent validity and high internal consistency reliability, and thus, its use could help improve measurement of person-centered family planning services.

Our results align with the underlying concepts that inform measurement of women’s decision-making power, decisional autonomy, and empowerment. Women’s empowerment, according to Kabeer, is understood to be a broader construct than either decision-making power or decisional autonomy, comprising three fundamental components: resources, the preconditions that enable one to exercise choice; agency, the process of defining one’s goals and acting on them; and achievement, the outcomes of implementing one’s choice [31]. Kabeer defines empowerment as the process by which individuals acquire the ability to make choices [31], which is rooted in Sen’s Capabilities Approach that focuses on granting individuals the capability to pursue the life that they prefer [32].

The first factor identified in the FP-ADM scale, “Bidirectional Exchange of Information” is related to the idea of resources in Kabeer’s empowerment framework, and reflects the concept of informed choice—a longstanding cornerstone of rights-based family planning programs. Informed choice is thought to be synonymous with autonomous decision making in family planning programs [33]. It is based on the idea that access to sound information is fundamental to decision-making, or a precondition to enable one to exercise choice, and that the choices relating to contraceptive use should reflect the decision maker’s values and preferences, with the client ultimately making the final decision about which method best meets their needs [34, 35]. With a goal of ensuring that informed choice is realized, approaches to contraceptive counseling have taken different paths [36]. On one end of the spectrum, one approach is to give clients information about all the methods in order to enable the woman to have a fully autonomous decision. Given the expansive range of contraceptive methods often available, and the nuances related to contraindications, possible side effects, and efficacy, ensuring a woman has complete information about every method is a goal that is arguably impossible to fully realize [34]. On the other end of the spectrum, another approach focuses on directive counseling, where providers give clients information on methods they deem to be most appropriate based on their own assumptions about what they think client may value. Operationalization of more directive approaches, especially with regard to recent provider preferences for long-acting and reversible contraception, raises concerns about reproductive justice and the potential for coercive programming [37]. A third approach lies in the middle, and that is shared decision making, which includes both provision of information, but also an exchange, to understand the client’s needs, values, and preferences, and to contribute one’s expertise to helping the client make the decision that best satisfies their needs [38, 39].

Whether women receive the contraceptive method of their choice is often used as an indicator of service quality. However, questions have been raised about whether a woman receiving the method of her choice actually reflects a process that results in her own decision being made [18] for the reasons discussed above. This is of increased concern in resource-poor settings where women may have limited a-priori information about contraceptive options and may be subject to overt and subtle forms of coercion [40, 41]. A 2012 Bellagio Consultation entitled “Contraceptive Choice in the 21^st^ Century–An Action Agenda” defined contraceptive choice as, “the fundamental right and ability of individuals to choose and access the contraceptive methods that meet their needs and preferences without either barriers or coercion.” [42] Our scale emphasizes that the bidirectional flow of information—most consistent with the shared-decision making approach to contraceptive counseling—is interrelated to the concept of autonomous decision, as are resources and agency.

The second dimension that emerged in the FP-ADM scale, “Empowered Choice,” emphasizes that women understand the notion of choice in the context of family planning services to be a separate, but related, pillar from the exchange of information, as resources are distinct from agency in Kabeer’s framework. Further, autonomy is a domain of empowerment relating to agency; agency, in turn, may be more closely related to decision-making power and more specific than the broader construct. Other frameworks also describe decision making as a domain of empowerment [43]. Ultimately, decision-making power or agency can be conceptualized as both a determinant and reflection of the outcomes of interest: empowerment and its related concept, autonomy–defined by Dyson and Moore (1983) as the ability to obtain and use information to make decisions about one’s own private concerns and those of intimates (i.e., independent, informed, un-coerced, free from control or influence) [44, 45]. With the focus on the verb "to decide", “empowered” or “with autonomy” becomes a qualifier of such action. Power is central to Kabeer’s framework, in that individuals who already possess the ability to make choices do not need to be empowered, or granted, the ability to make choices. However, in the context of family planning, providing women with information enables women to decide which method is best for them; however, they may not have the intrinsic power to realize their choice without the support of a provider. Therefore, the “empowered choice” factor in our scale reflects both of these fundamental aspects.

The third aspect of empowerment as defined by Kabeer relates to achievements, or the tangible outcomes indicating that empowerment has successfully occurred [31]. In the realm of contraceptive counseling and service provision, informed choice may be associated with increased satisfaction and method continuation [34]. We did not see the same positive association between contraceptive use and method satisfaction in India as in Argentina and Ghana, although there was still a positive association with overall visit satisfaction and the FP-ADM scale. It is possible this could reflect limited options for women to choose from within the Indian context coupled with very high use of permanent methods—particularly, female sterilization [46, 47]. Irreversibility of the method could plausibly explain the lack of association between current contraceptive use and method satisfaction, and between using a woman’s preferred method and intention to continue until a pregnancy is desired. Further, a high degree of social desirability bias is associated with sexual matters including use of contraception [48]; such an explanation would be consistent with other outlying results in our survey data for India.

A strength of this study is that it includes data from a large sample of women in three diverse low- and middle-income countries. Differences in the facility-based sample in Argentina versus the community-based sample in Ghana and India appears to contribute to the variation observed in population characteristics between these study settings (i.e., a higher proportion of respondents were pregnant in Argentina than in the other countries, and the content of their visit was much more specific–method provision and method-specific counselling) than in the other two settings where the last family planning encounter was more likely to be community-based rather than facility-based. While this could be construed as a limitation, we believe that it this is a strength because we have validated the scale in both facility and community-based samples.

However, there are some limitations to this study. We do not have prospective data on contraceptive use; thus, we cannot assess predictive validity. Our study is subject to recall bias, as women were asked about their most recent family planning visit, but the timing of that visit was not bound to a specific window. The validation study for the IQFP scale in India also asked women about their most recent contact with a family planning service provider, but restricted the analysis to women who have received family planning services within the last year [25]. We believe that there are benefits and drawbacks to both approaches. While excluding women who received family planning services more than a year ago may limit recall bias, leaving the timeframe open enabled us to include women who have been using their method for longer. To explore the extent to which recall bias may influence our results, we performed a sensitivity analysis in which excluded women who were using a method with a viable duration longer than one year (sterilization, intrauterine devices, and implants) to assess any differences in the results. This analysis revealed no substantive differences in the findings. In our study, women were included if they only received written information from a family planning provider. While it is unlikely that providers who are only giving written materials are likely to be providing the highest quality of care, it is a reality that many women in our study countries have faced–as evidenced by our data. Further, the majority of family planning visits across all study settings did not result in method provision. There are several reasons why few family planning visits may not have resulted in a method being provided, including women opting for natural methods, women who are currently using a method and are interested in learning more about their current method or are interested in switching methods, women who receive information but want to decide on a method after taking some time to think about their preferred method or discuss it with their partner, women who use condoms, or women who do not want a method. In the communities where the study took place in Ghana and India, it is common for women to receive general family planning information from a frontline, community-based health worker who will require a woman to have a follow up visit or to obtain a method from another provide or source, should she require a method to be provided. In Argentina, provision of family planning information is a required component of all reproductive and maternal health services. We argue that our scale is still relevant for women who did not receive a method during their most recent family planning visit, as autonomous choice is also important for women who choose not to use a method of family planning. In the validation of the IQFP scale in India, participants were also not required to have received a method during their last family planning visit [25].

Finally, our results may be influenced by some endogeneity due to reverse causation; women who are generally more satisfied with their method after using it may be more likely to have positive memories of their most recent FP visit.

A validated tool to assess decisional autonomy is a useful addition to the armamentarium available to assess women’s’ empowerment and person-centered care in the context of sexual and reproductive health and rights. Data collected using the FP-ADM scale may contribute to population-level measures of decisional autonomy in family planning services, including the proposed new indicator focused on decisional autonomy and contraceptive choice, “proportion of women of reproductive age who make their own decisions about if, when, and how many children they want.”

## Supporting information

S1 Checklist Inclusivity in global research checklist. (DOCX)

## Abbreviations

EPMM: Ending Preventable Maternal Mortality
FP-ADM: Family Planning Autonomous Decision-Making Scale
IQFP: Interpersonal Quality of Care in Family Planning Scale
MADM: Mothers Autonomy in Decision-Making Scale
